# Microencapsulation of Probiotics for Food Functionalization: An Update on Literature Reviews

**DOI:** 10.3390/microorganisms10101948

**Published:** 2022-09-30

**Authors:** Maram Sbehat, Gianluigi Mauriello, Mohammad Altamimi

**Affiliations:** 1 Department of Nutrition and Food Technology, An-Najah National University, Nablus P.O. Box 7, Palestine; 2 Department of Agricultural Sciences, University of Naples Federico II, 80055 Portici, Italy

**Keywords:** functional food, probiotic food, probiotic cell preservation

## Abstract

Functional foods comprise the largest growing food category due to both consumer demands and health claims by manufacturers. Probiotics are considered one of the best choices for meeting these demands. Traditionally, the food vehicle for introducing probiotics to consumers was dairy products, and to expand the benefits of probiotics for a wider range of consumers, the need to use other food items was essential. To achieve this goal while maximising the benefits of probiotics, protection methods used during food processing were tackled. The microencapsulation of probiotics is a promising methodology for achieving this function. This review highlights the use of the microencapsulation of probiotics in order to functionalise food items that initially were not considered suitable for probiotication, such as baked products, or to increase their functionality such as dairy products. The co-microencapsulation of probiotics with other functional ingredients such polyphenol, prebiotics, or omega-3 is also highlighted.

## 1. Introduction

In 2001, the World Health Organization defined probiotics as “live microorganisms which when administered in adequate amounts confer a health benefit on the host”. In 2014, the definition was reworded to be more grammatically correct, as “live microorganisms that, when administered in adequate amounts, confer a health benefit on the host” [1].

The term probiotics includes strains belonging to several genera of bacteria and yeasts, such as *Lactobacillus* (this genus was recently reclassified in 25 genera by Zheng et al. [2]), *Bifidobacterium*, *Streptococcus*, *Enterococcus*, *Lactococcus*, *Bacillus*, *Escherichia*, and *Saccharomyces*. These microorganisms can naturally be found in fermented foods obtained by using natural starter cultures. In addition, probiotics are available in the market in the form of supplements or as probioticated foods. The interest of probiotics and its introduction to foods massively increased during the past few years due to reported evidence about probiotics positive effect on human health. Probiotics promote health statuses and play an essential role against the colonization of pathogenic microbes in intestines by the production of antimicrobial compounds, increase gut integrity by stimulating mucus production, improve enzymes formation, regulate the composition of gastrointestinal (GI) microbiota, and act as immunity modulators. Currently, investigations about the importance of GI microbiota widely and intensively increased. Moreover, this demonstrates the major role of probiotics and GI microbiota composition in the progression of many diseases and disorders, such as obesity, allergies, diabetes, inflammations, inflammatory bowel diseases (IBD), cancer, infectious diseases, and even neurodegenerative diseases [3].

This has increased its global selling price from USD 42.55 billion in 2017 to USD 94.48 billion by 2024 [4]. All figures are dominated by Asia and the Pacific, whereas rules governing health claims in Europe have resulted in a modest growth rate of probiotic types of foods. Moreover, economic analysts expect an increase in demand for functional foods, especially during the COVID-19 pandemic. This increase is due to consumer’s awareness about the benefits of probiotics and its effect on health and, particularly, immunity boosting, and their tendency towards food in general.

To achieve improved utility, we need to be sure that probiotic products take in account the differences in needs amongst all population categories and the consumption of that product in a specific market. For example, (1) if we probioticate bread, we should consider the celiac disease population and gluten-free dieters, (2) milk products probiotication should consider lactose-intolerant patients, so we think about the probiotication of alternatives suitable for this category such as almond or soy milk and (3) take into consideration the coating agents in the case of vegan consumers. Moreover, as expected, the demand for plant and animal proteins excluding the meat, poultry and seafood will increase, so the probiotication of food should take in account these economic expectations during the coming years.

To obtain the maximum health benefits claimed about probiotics, the number of viable probiotic cells should not be lower than 10⁶–10⁷ CFU/mL when ingested according to the Food and Drug Administration and World Health Organization [5]. Unfortunately, the number of probiotics ingested are affected by numerous physiological factors, including (1) chemical (low pH, gastrointestinal conditions, food matrix properties, processing, and storage conditions) and (2) physical factors that affect the adhesion and colonization of probiotics in intestines such as the rapid transit time [6].

To overcome these barriers, scientists applied several strategies to improve probiotics viability, including nanoparticles, polymer gels, and microencapsulation to protect sensitive probiotic cells against harsh conditions in gastrointestinal tracts and during storage and to improve the adhesion to mucosal lining. In particular, the microencapsulation refers to physicochemical processes to entrap an active compound or cell in a material in order to improve its functionality. In 1955, the chemist Barrett K. Green received a patent for the process of microencapsulation [7]; at first, it was for typing paper and then it widened to include pharmaceuticals and food industries.

The aim of this review is to summarize scientific investigations, from 2015 to date, that dealt with the application of microencapsulated probiotics in different food matrices in order to present how microencapsulation can protect probiotics against harsh conditions.

## 2. Food Probiotication with Microencapsulated Cells

Despite some problems facing the market of probiotics around the world, it is still growing substantially due to reasons that are mentioned in the Introduction, in addition to high investments on the research and development sector and increase in competition between companies. To keep up with the situation, the probiotication of food also increased. Interestingly, the probioticated food is not only limited to basic dairy products, but it also includes baked goods, ice creams, desserts, fruit and vegetable juices, jams, olive paste, meats, and even some traditional recipes such as koozh and doogh [8,9,10,11,12,13].

According to probiotic market analysis, bacterial probiotic strains are used more than yeast, especially in food industries. The main bacterial strains used for food probiotication belong to several species of *Lactobacillus* and *Bifidobacterium,* and the most popular yeast species is *Saccharomyces cerevisiae (boulardii)* [14].

Microencapsulation refers to physicochemical processes that entrap an active compound or cell in a material in order to improve its functionality. The most investigated purpose for the microencapsulation of probiotic cells is to protect against gastrointestinal conditions [14]. However, the effect of the food matrix and processing conditions on the survivability of microencapsulated probiotic cells should not be neglected. Indeed, the shelf life of a probiotic food is mainly based on the dropping kinetics of the alive probiotic population in the food product [1].

### 2.1. Baked Goods

As previously discussed, to guarantee the delivery of the probiotic to the largest possible number of populations, the product should be familiar and take in account the market analysis of that product. Globally, bread is considered as a primary food due to its variety and convenience. By the probiotication of bread, we assure the daily delivery of probiotics to consumers, while biscuits and cakes are consumed as snakes amongst all population classes. Presently, the probiotication of bread and other baked goods and pastries is limited due to severe injurious effects of high-temperature treatments, aerobic conditions that are unsuitable for probiotic survival, and long storage time in some categories, in addition to chemical reactions occurring within food matrix.

Microencapsulation approaches that increase the viability of probiotic cells in bakery products to either protect it against disturbing conditions and assure the stability under storage conditions during the product’s shelf life are deemed the best.

As a result, the number of published studies about the probiotication of baked goods has been low in the past five years [15,16,17,18,19,20]. Generally, the incorporation of microencapsulated probiotic cells in bakery production was covered by three approaches: (1) in cream or filling materials, (2) addition to cake mix or dough, and (3) the distribution of powdered cells on the surface of the dough before baking.

Interestingly, Zhang et al. (2018a) [17] studied the effect of dough size and the modification of time-temperature combinations on the basis of the gelatinization of starch to improve viability of probiotics with a minimal alteration in the quality of the final product.

Practically, the harvested *L. plantarum* P8 cells were re-suspended in reconstituted skimmed milk (RSM), gum Arabic, maltodextrin, and inulin solutions and then dried using a vacuum freeze-drying technique. The bacterial cells obtained were incorporated into bread by three approaches: (1) suspension with a dough ingredient, which was UHT skimmed milk, (2) cell powder was thoroughly mixed into dough as the last item, and (3) distribution on the surface of the dough ball. The viable count of *L. plantarum* in bread before and after baking was determined at 175 ℃ for 6 min or at 100 ℃ for 15 min.

In general, the baking process significantly reduces the viability of microencapsulated cells. However, the results showed the higher viability of microencapsulated probiotic cells in cases of shortening baking times with higher temperatures, with little effect on the size of the dough during controlled times. Moreover, the viability of probiotic cells was higher in the crust than that in the crumb with respect to differences in water content and, during storage, alterations in titratable acidity. These findings correspond with Seyedain-Aradabili et al. (2016) [15], who found a higher survival rate of microencapsulated probiotics in hamburger buns more than white loaf because of the shorter time for heat exposure.

On the other hand, the viability of free *Lactobacillus plantarum* was the highest at same baking conditions when applied onto the surface of dough before baking, as shown by Zhang et al. (2018b) [18]. This obviously related to lower moisture contents. On the other hand, the incorporation of free probiotic cells by mixing did not show any protective effects against baking temperature, which is explained by the higher exposure of cells to moisture and higher heat transfer.

With respect to microencapsulated probiotic cells, Thang et al., (2019) [19] studied widely different types of encapsulating materials that are identified as the best carriers to supplement bread with *L. acidophilus*. Four coatings treatments were applied: Alginate (ALG), ALG+ maltodextrin (AM), ALG+ xanthan gum (AX), and ALG+ maltodextrin+ xanthan gum (AMX) using the emulsifying method and supplemented in bread. They found that the best results were obtained when encapsulation treatments contained xanthan gum (AX and AMX) with an encapsulation yield of 92.37% and 92.9%, respectively. With the presence of maltodextrin as a coating material, AM and AXM conferred higher protections against high baking temperatures at 185 ℃ for 13 min compared to ALG and AX. However, with the combination between three agents, AMX provides the highest survivability of encapsulated *L. acidophilus* during storage as well as under simulated gastrointestinal conditions.

During storage, the viability of microencapsulated probiotics in bread slightly increased during the first two days [19], which was related to the consumption of bread as a substance for probiotic growth, and then its viability decreased for storage conditions. Contrarily, in Zhang et al.’s (2018a) [17] study, they found no significant increases in probiotics viability over the first two days of storage and a higher survival rate until day-5. They explained these results to consider the first two days during the recovering phase for injured cells and then examined regrowth. The differences in results may be related to the type of bread, baking, and storage conditions or even due to different probiotics and coatings used.

In the case of cake production, no surviving probiotics were reported when the microencapsulated cells were incorporated within the cake mixture. On the other hand, the integration of microencapsulated probiotic cells to cream or filling material exhibited a higher survivability [16,20] because of the stability of hydrophobic coatings leads to the high protection of cells.

### 2.2. Dairy Products

#### 2.2.1. Milk

The biggest challenge that faces the production of probiotic milk is preserving the desired fluidity and preventing the proteolysis of milk proteins that would turn it into a fermented product or, in other specific words, a ‘yogurt-type’ product. The addition of free probiotic cells alters the texture and the flavor of ordinary milk.

García-Ceja et al. (2015) [11] scrutinized the viability of microencapsulated *Lactobacillus acidophilus* and *Lactobacillus reuteri*, with alginate (ALG) or alginate-chitosan (ALG/CHI), in both milk and blackberry set-style yogurt. They found that cells encapsulated with ALG/CHI exhibit improved protection and improved the viability of microencapsulated probiotic cells to meet the recommended count of probiotics more than 10⁷ CFU/g, even after 30 days of refrigerated storage in both milk and set-style yogurt. On the other hand, alginate-coated beads maintained a recommended probiotic count for both lactobacilli strains after 30 days only in blackberry jam set-style yogurt. Despite the nature of *L. reuteri*, it maintained the recommended count when coated with alginate only in set-style yogurt, while the survivability with the ALG/CHI coating was 9.1 and 8.1 logs in both set-style yogurt and milk, respectively. This may be related to the difference in pH and composition of tested foods; *L. reuteri* has, comparatively, a weak proteolytic activity. Finally, they deduced that milk and blackberry jam set-style yogurt improve microcapsules’ stability.

From a sensorial point of view, the incorporation of microencapsulated probiotic cells has different criteria. There was no significant alteration in the sensory evaluation of blackberry set-style yogurt due to the presence of seeds in jam [11]. Otherwise, the texture affected by the presence of beads in milk without negative changes in flavor. However, spray drying microencapsulated probiotic cells can solve the differences that may occur in physicochemical properties and sensorial attributes [21].

#### 2.2.2. Fermented Milk and Yogurt

The incorporation of microencapsulated probiotic cells in plain yogurt and fermented milk was previously studied [21,22,23,24,25,26,27,28,29,30,31,32,33,34,35,36] to prevent a significant loss in the viability of probiotics because of acidic pH in such products.

Both Dimitrellou et al. (2016) [21] and Patrignani et al. (2017) [29] studied the integration of probiotics to fermented milk with two different techniques in an attempt to acquire the therapeutic level of probiotic cells in food and, additionally, to prevent the occurrence of unpleasant textural and sensorial changes.

The survival rate of microencapsulated *Lactobacillus casei* in fermented milk reported to be significantly higher than that of free cells, starting with the third week of refrigerated storage. After four weeks, the survival rate of *L. casei* was 17.8% and 9.5% for both microencapsulated and free cells, respectively, and still was higher than the recommended count to implement the therapeutic effect of probiotics [21]. The same results were obtained by Patrignani et al. (2017) [29], who reported a higher viability in microencapsulated *Lactobacillus paracasei* A13 and *Lactobacillus salivarius* CET 4063 in fermented milk under storage conditions for more than 30 days, with lower pH changes.

In the same vein, Hashemi et al. (2015) [13] support the previous results. They studied the development of probiotic Doogh (a traditional Iranian fermented milk drink) with *L. plantarum* LS5 and inulin as prebiotic. Additionally, the presence of inulin improves the survivability of both free and microencapsulated cells, without any significant alterations related to sensory attributes up to 22 days of storage.

In Table 1, main information about studies on the incorporation of yogurt and cheese with different microencapsulated probiotic strains is provided. In particular, we report the utilized probiotic strains, method, and material for the encapsulation; storage conditions; cell survival during the storage period, and effects on sensory characteristics and pH.

#### 2.2.3. Ice Cream

As stated previously, probiotics should be delivered by using familiar products. Globally, ice cream is one of the most popular nutritious and tasty foods amongst all population classes, regardless age, due to its various flavors and charming texture. Therefore, we can consider ice cream as a proper vehicle for probiotics. In such cases, the biggest challenges are (1) to preserve the quality of ice cream product or improve probiotic ice cream, at least with minimal sensorial changes, and (2) improve the protection against low temperatures in addition to other harsh conditions. Therefore, there are a few studies worked on improving probiotic ice cream products [8,39,40,41].

In study of Afzaal et al. (2019b) [39], they investigated the effect of encapsulation on the stability of *L. acidophilus* ATTC-4356 when inoculated in ice cream. Alginate- and carrageenan-encapsulated probiotic cells were inoculated in an ice cream mixture and incubated at 40 °C until the pH reached 6.5 and then they were frozen at −4 to −5 °C and stored at −20 °C for 120 days. Immediately after shock freezing, a high death rate was reported in encapsulated *L. acidophilus* because of cell damage that related to the formation of ice crystals, but it was still lower than that reported for free cells. However, this loss of encapsulated cells declined during storage. Specifically, the viable count of alginate- and carrageenan-encapsulated probiotics decreased from 9.9 and 9.8 log CFU/mL at 0 day to reach almost 8.9 and 8.5 log CFU/mL after 120 days of storage, respectively. Moreover, the behavior of capsules when exposed to simulated gastric and intestinal fluids positively affected its survivability under such harsh environments. Unpleasantly, the sensorial characteristics of ice cream (color, texture, taste, appearance, and overall acceptance) were significantly affected by the inoculation of products with probiotic cells in both forms, either free cells or encapsulated.

In contrast, Kataria et al. (2018) [40], who encapsulated *B. longum* CFR815j in alginate and starch, reported slight but not significant differences in the sensorial characteristics of probiotic ice cream. Indeed, the mixing of alginate with starch in encapsulation showed efficient protections and recovery for probiotic cells.

The same experimental design was performed by Afzaal et al. (2020) [41] with different encapsulating agents, calcium alginate and whey protein concentrate (WPC), in order to improve the survivability of *L. casei* when injected in ice cream. Similarly, the losses of viable cells for ALG- and WPC-encapsulated probiotic cells were only 0.55 and 1.13 log CFU/mL, respectively, while a 3-log reduction was recorded in the case of non-encapsulated cells. Moreover, they observed an increase in the viscosity of ice cream incorporated with ALG- and WPCI-encapsulated *L. casei* compared with the control with viscosity values at 250, 300, 270, and 220 cp over 80 days of storage. This increase could be related to presence of alginate and WPC, which bind to free water during storage.

Spigno et al. (2015) [8] specified his work for a very important and new criteria: the development of new powdered ice-cream formulations with *L. paracasei* encapsulated in alginate and maltodextrins (formula A) or alginate and inulin (formula B). The results showed no effects with respect to formula types on processing yields (58–66%) nor water activity (<0.3). Nevertheless, the incorporation of microencapsulated probiotic cells would be attainable but with higher powder water activity (>0.4) and lower process yields (51%).

#### 2.2.4. Cheese

With the wide range of cheese types, processing, and ripening conditions, probiotic strains were chosen and microencapsulation techniques were used, resulting in an estimation of the effectiveness of fortifications with respect to cheese with microencapsulated probiotics [42]. However, cheese has advantageous properties that make it the best probiotics carrier compared to other fermented dairy products, including its buffering capacity, relatively high fat content, and high-density matrix, which confer additional protections during digestion and transit [43].

Sharifi et al. (2021) [44] studied the fortification of Iranian white cheese with encapsulated L. plantarum. They found a significant decrease in free L. plantarum compared to those encapsulated with whey protein isolates and gum Arabic during 61 days of storage. However, they obtained the highest survival rate for samples of L. plantarum, which co-encapsulated with phytosterols.

Mudgil et al. (2022) [45] using Chami (traditional soft cheese) has shown that the maximum survival rate of *Pediococcus pentosaceus* when exposed to simulated gastrointestinal conditions was observed when probiotic bacteria were encapsulated with camel milk protein (98.6%), followed by wheat-starch-encapsulated cells with a viability rate of 70.7%. Moreover, during 9 days of storage at 4 °C, no significant reduction in viable cells was observed in cases of using camel milk protein as an encapsulating material, while significant differences were observed after 3 days for free cells and wheat-starch-encapsulated cells.

However, studies discussing the fortification of cheese with encapsulated probiotics should not exclude the sensorial evaluation of such products and the effect of fortification on cheese characteristics. On the same line, Kavas et al. (2021) [46] concluded insignificant changes in the sensory traits of goat cheese after 180 days of storage when L. lactis and B. longum encapsulated with alginate, fructooligosaccharide+ alginate, and inulin+ alginate separately. In a complementary line, the viability of *L. paracasei* and *B. longum* was preserved at a higher level in goat-cheese samples obtained with the addition of prebiotics to microcapsules throughout 180 days of storage at 4 °C [47].

With respect to the same topic, Mukhtar et al. (2020) [48] reported that the loss of viability of encapsulated L. acidophilus was noticeably lower compared to free cells in mozzarella cheese, while supplemented cheese samples, with free and encapsulated cells, possessed improved sensory attributes compared to the control sample.

### 2.3. Fruits and Vegetable-Based Products

Fruits and vegetables in general contain high levels of functional components naturally, such as fibers and prebiotics, vitamins, minerals, and antioxidants, which were reported to confer special properties to this type of foods.

Many scientists investigated the development of probiotic fruit- and vegetable-based products, including juices [11,49,50,51,52,53,54,55,56,57] (reviewed in Table 2), jam [9,11,58], jelly [59], fruit powder [55,60], and vegetable juice [10].

Gonzàlez-Cuello et al. (2018) [58] studied the possibility of incorporating an encapsulated *L. bulgaricus* as a probiotic into low-calorie tree tomato jam. This type of product was developed to prevent health problems by partially replacing sucrose with stevia (*Stevia rebaudiana*) as a sweetener and the addition of *Aloe vera* (*Aloe Barbadensis Miller*) to improve health functionality of jam. Unfortunately, during 20 days of storage, the viable count of encapsulated probiotic cells, lower than 3.6 × 10^3^ CFU/mL, was not high enough to reach the therapeutic level.

The incorporation of free probiotic cells to peach nectar exhibited unpleasant characteristics in the final product; precipitated cells were negatively altered the appearance of the nectar, and undesirable fermented flavour was developed and affected the texture of the nectar, after 20 days, due to the pectolytic activity of *Lactobacillus acidophilus* [11]. On the other hand, ALG/CHI encapsulated with *L. acidophilus* and *L. reuteri* maintained the recommended level when incorporated into both peach nectar and blackberry jam, even after 30 days storage at 5 °C.

Bora et al. (2019) [60], who was trying to produce a probioticated freeze-dried banana powder, performed an interesting study. They found that the encapsulation of *L. acidophilus* and *L. casei* with the combination of whey protein isolates (WPI) and fructooligosaccharide (FOS) has a significant protection effect over 30 days storage at 4 °C, averaging 8.57 and 7.61 log CFU/mL, respectively. Moreover, sensorial characteristics of the product were not significantly altered after probiotication.

There was a single study that studied fruit jelly as a vehicle of probiotic cells. In the work of Talebzadeh and Sharifan (2017) [60], they studied the incorporation of ALG/CHI-coated *L. acidophilus* LA-5 in fruit jelly and investigated its effect on survivability during storage and under harsh conditions, pH, syneresis, and on the sensorial characteristics of jelly. They found a significant protecting effect (*p* < 0.05) of ALG and ALG/CHI coatings with viable counts at 7.04 and 8.72 log CFU/g, respectively, after one month of storage at 7 °C. In contrast, a dramatic decrease in survivability, below 5.28 log CFU/g of non-encapsulated cells, was observed. Moreover, a considerable increase in syneresis for all groups of probioticated jelly was observed. Indeed, alginate beads exhibited the lowest syneresis, 2.52% at 7 °C after 6 weeks, related to binding with water, which led to an increase in viscosity, while syneresis formation rates were the highest for ALG/CHI jelly. Pleasantly, the texture, flavor, odor, and overall acceptance of probiotic fruit jelly were significantly accepted without expected sandy textures, despite an unsuitable color and appearance compared to the control sample.

For vegetable-based products, Naga Sivudu et al. (2016) [10] studied the microencapsulation of several strains of probiotic bacteria and yeast to produce probiotic tomato and carrot juices. The viability losses in both juices ranged between 1.6 and 5.2 log CFU/mL and 0.8 and 2.7 log CFU/mL for free cells and microencapsulated ones, respectively. Moreover, the viable count after 6 weeks of storage at 4 °C decreased slightly, a 0.1–1.9 logs reduction, for microencapsulated cells and a significant loss was obtained, at 3.2–4.8 logs reduction, for free cells. Generally, microencapsulated cells survived better than free cells. Specifically, the best viability results were recorded for *Lysinibacillus sphaericus* and *L. casei* in both tomato and carrot juice. Expectantly, texture and appearance were negatively altered, and swallowing became difficult because of beads’ presence.

Potato chips were also studied to be probioticated with microencapsulated *Lactococcus lactis* KUMS-T18 (Kiani et al., 2021) [61] using a traditional Iranian dish, tarkhineh. The bacterial tolerance to GI conditions, low pH 2.5 for 3 h’s and 0.3% bile salts for 4 h’s, was also studied, in addition to the sensory evaluation of the final product. It is noteworthy that encapsulated probiotic showed high tolerance to GI conditions, and probiotic potato chips received acceptable sensory evaluation scores compared to the control during 4 months of storage at 4 °C.

### 2.4. Other Products

Food probiotication was not limited to daily used products, as it also included other secondary items and dishes. For instance, Alves et al. (2015) [12] formulated an olive paste to deliver the encapsulated *L*. *plantarum* 33. The viability of *L. plantarum* was reduced by 2- and 3-log cycles after 30 days of storage at 22 °C for encapsulated and free cells, respectively, but this reduction lowered probiotic levels below the therapeutic count threshold. On the other side, when samples were stored at 4 °C for 30 days, the survivability of both encapsulated and free cells maintained the therapeutic level with a higher score for encapsulated cells. In addition, bacterial cells were exposed to high temperatures of 72, 85, and 90 °C, and interestingly, encapsulated cells showed a significant resistance against all heat treatments. Moreover, microencapsulation significantly increased the viable count of probiotics cells with 1.37 × 10⁷ and 7.9 × 10⁶ CFU/g under SGJ and SIJ, respectively. No significant changes were observed in all sensory attributes except for flavor, and probiotic-induced off-flavors may be related to encapsulating agents.

Almond milk, in addition to its high nutritional value, is considered as one of the most common alternatives for cow milk consumed mainly by vegan and lactose-intolerant populations. Its probiotication was also studied, as a powder, with microencapsulated *L. plantarum* ATCC8014 [62]. The results revealed a significant reduction; the viable count was below the recommended level when stored at 22 °C for 8 months. However, survivability was higher than therapeutic levels over 8 months at 4 °C and up to 6 months at 22 °C. From a nutritional point of view, the content of saturated, mono-unsaturated, and poly-unsaturated fatty acids profiles was not significantly affected by storage time and temperature, while some minerals such as magnesium and manganese contents were higher than that in raw almond.

Endophytic lactic-acid bacteria strains KCC-42 and KCC-41 were microencapsulated and incorporated into radish and cabbage kimchi [63,64], respectively. Encapsulated cells showed a fermentation activity with higher pH decreases in fermented cabbage kimchi due to the higher production of organic acids. In addition, a higher survivability at 6.53-log CFU/mL, up to 12 days and stabilized up to 21 days, was observed, while when compared to non-encapsulated cells, the survivability was only 4.35 log CFU/mL. No significant effects were observed in sensory characteristics between two samples of cabbage kimchi inoculated with either encapsulated lactic acid bacteria or non-encapsulated cells. The same results were obtained when strain KCC-42 was incorporated into radish kimchi [63].

In contrast, microencapsulation showed no additional protections, compared with free *L. reuteri* probiotic when incorporated into soy beverage [51]. The results revealed less than a 1 log CFU/mL reduction in viability after 8 weeks of storage at 4 °C and 8 °C. Indeed, post-acidification effects of probiotics were significantly observed, particularly at 8 °C for encapsulated cells.

In the study of Witzler et al., (2017) [65], they developed a diet probiotic lozenge with *Enterococcus faecium* CRL183. Microencapsulation was performed by coacervation and extrusion. Viability maintained the desired level after 273 days of storage in the case of coacervation, while extrusion could not protect *E. faecium* even after a few days.

A popular snack also joined the microencapsulation world: chocolate. Marcial-Coba et al. (2019) [66] studied the incorporation of *L. casei* and *Akkermansia muciniphila* in dark chocolate. The results showed no significant reduction in encapsulated *L. casei* during storage at 4 °C and 15 °C for 60 days. On the other hand, A. muciniphila exhibited no significant losses during the first 30 days of storage at both temperatures, while the significant reductions were recorded after 60 days of storage at 4 °C and 15 °C, with 0.63 ± 0.05 and 0.87 ± 0.05 log 10 CFU/g reduction, respectively. Pleasantly, microcapsules did not significantly alter the acceptance of dark chocolate but still scored a lower point in sensory evaluation tests because of the presence of calcium chloride residues. The same observations obtained from previous studies on milk chocolate were reviewed by Gadhiya et al. (2015) [67].

Additionally, Rajam et al. (2015) [68] developed a new type of probiotic noodles by using microencapsulated *L. plantarum* MTCC 5422. The microencapsulation process performed by freeze-drying with wall material FOS+ WPI and FOS+ denatured WPI. The results revealed that the microencapsulation of probiotic cells with FOS+-denatured WPI conferred superior protection for cells after freeze-drying, during the storage of noodles, and under simulated gastrointestinal conditions. However, all microencapsulation systems showed at least a 98% survival rate. Importantly, the presence of microcapsules affected the moisture content and cooking time for both fresh and dried noodles due to water uptake by coating materials, especially for denatured WPI that has a high-water holding capacity and relatively lower gluten content without significant effects on sensory attributes, except for colour. Surprisingly, the viability of encapsulated probiotic cells in fresh noodles after cooking was 62.42%, which is higher than dried noodles. This result is explained by the high drying temperature and lower viability of probiotic cells in dried noodles, 80.29% compared with 93.63% in fresh noodles, before cooking. The same results were obtained by Kalkan et al. (2020) [69], who prepared Turkish noodles supplemented with microencapsulated *Bacillus clausii* and vegetables to enhance flavour. In order to add vegetables, the control sample had the shortest cooking time because of same pre-mentioned reason: the content of gluten. Unfortunately, the viable count of microencapsulated cells decreased in every processing step until it reached 5.02 to 5.1 log CFU/g after cooking, which is lower than therapeutic recommended level of probiotics.

## 3. Co-Microencapsulation of Probiotics

Although the main purpose of microencapsulation is to enhance the food functionality, it was suggested that incorporating materials such as prebiotics (inulin and mannitol) may confer further functionality [70]. Other materials such as antioxidants (quercetin) were also suggested. Lipids including α-linoleic acid were shown to increase encapsulated probiotics through in vitro digestion. A two-fold survivability rate was obtained when *L. casei* was co-microencapsulated with omega-3 oil [71]. Polyphenols, possessing prebiotic effects, were also evaluated as co-material to enhance the survivability rate of *L. casei.* The antioxidant activity did not change, and cell count slightly decreased from 9.39×10^9^ CFU/g to 4.41×10^9^ CFU/g [72]. Co-microencapsulation is a promising approach to functionalise food and to enhance probiotics viability [73]. The approach is widely open for combinations between probiotics and other active compounds that were proven to be effective prebiotics.

Although the symbiotic effect of many materials was extensively studied, few researchers have evaluated the symbiotic effect of co-microencapsulated probiotics. Symbiotic materials can have a complementary or a synergistic effect when co-microencapsulated with probiotics [74]. As internal environments may serve as a hostile medium for external probiotics, symbiotic co-microencapsulation will provide protection and further nourish specific probiotics. Such techniques assist in modulating the gut microbiota and improve food functionality. There are three categories so far that work as co-microencapsulated materials:

1. Omega-3 and GABA;
2. Soluble dietary fibres;
3. Phytochemicals.

It should be noted that probiotics–prebiotics combinations can be strain-specific; moreover, selecting the right co-microencapsulation method should be considered in order to maximise benefits [74].

Novel compounds that provide activities beyond being prebiotics are under investigation: for example, antidiabetic compounds that inhibit alpha-amylase activity [75], lactoferrin, which has multifunctions in humans [76], and bacteriocins, which act as antipathogenic agents [77].

Co-microencapsulation using nanoparticles was also evaluated. Huq et al. (2017) [78] found that the addition of cellulose nanocrystal (CNC) and lecithin in alginate microbeads (ACL-1) improved the viability of *L. rhamnosus* during gastric passage and storage. Microencapsulation using alginate and chitosan nanoparticles was found not only to enhance probiotics *Escherichia coli* Nissle 1917 viability but also reduced *Campylobacter jejuni* growth by 2 log CFU [79]. The incorporation of nanoparticles in microencapsulation methods will provide stability during the harsh environment of GIT, increase solubility and the bioavailability of materials, and sustain probiotic release; however, nanoparticles’ safety is debatable [80]. A highlight on contemporary and emerging single-cell encapsulation strategies using nanocoatings for individual probiotic cells is found in Centurion et al., (2021) [81]. The field of nanoparticles and its applications in probiotic encapsulation will progress, and novel biomaterials will join in the near future.

## 4. The Market of Microencapsulated Probiotics

**Figure 1:**
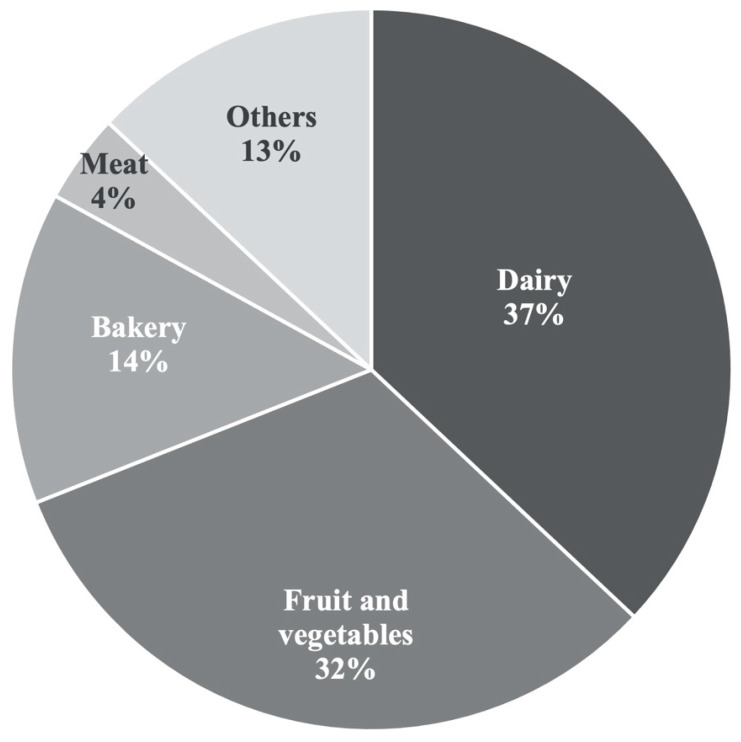
Percentage distribution in the literature of papers dealing with probiotic microcapsules application in different food categories in the period 2015 to date.

Generally, microencapsulation is a fast-growing market that was estimated to be worth USD 8.4 billions in 2021, with a 9.8% annual growth rate and with carbohydrate as a leading carrier material in the food industry [82]. On the other hand, probiotics are the fastest growing functional food, especially in the Asia-Pacific region where 60% of the global population is presented. Gargi Dey (2018) [83] reviewed dairy and non-dairy probiotic foods and listed the main probiotic yogurt and milk, fruit juices, cereal-based products, and other types of products found in the market with free and microencapsulated probiotic in different countries: USA, Switzerland, Canada, India, New Zealand, Sweden, and others. However, the authors did not discriminate probiotics in free or microencapsulated forms, and by looking at the cited references, readers can distinguish the two different situations. The percentage distribution in the literature of papers dealing with probiotic microcapsule applications in different food categories are depicted in Figure 1, showing that the incorporation of microencapsulated probiotics into food was distributed between different food categories with different percentages. Indeed, the results were satisfactory compared with a similar analysis performed by De Prisco and Mauriello, (2016) [14], which reported that 49% of papers, in the decade 2006–2015, focused on milk-based products, 28% focused on fruit and vegetable-based products, 13% focused on meat-based products, and 10% focused on bakery products. Interestingly, while percentages of probioticated dairy products and meats decreased, from 49% to 37% and from 13% to 4%, respectively, bakery products, fruits, and vegetable-based products increased, from 10% to 13% and from 28% to 32%. Unfortunately, it is not very clear what impacts have been caused with respect to the market of this plethora of scientific papers on probiotic microencapsulation for food functionalization.

Nevertheless, investigations about the probiotication of food should be continued and should be commercially available in considering variations and differences amongst population groups. In this context, the main issues that should be taken into consideration are the price of the materials, R&D costs, and the technique used with additional process costs.

## 5. Conclusions

Investigations carried out about the incorporation of encapsulated probiotic into food are very important. Microencapsulation has been proven to enhance the survivability of probiotics in different food items. The percentages of new types of probioticated products were improved, and studies were not limited to dairy products, which is considered progress in the right direction. However, further investigations should be applied to focus on other stable foods that are also consumed on a daily basis, especially bakery products and cereals, and other products targeting patients with chronic conditions. In addition, more attention should be paid to less frequent or special cases, and a survey on the use of microencapsulated probiotics for food functionalization at an industrial level is highly desirable.

## Figures and Tables

**Table 1:** Incorporation of microencapsulated probiotics into yogurt and cheese and its effect on viability during storage.

Probiotic Strains	Encapsulation Facts	Storage Conditions	Viability (At the End Storage)	Capsule Size (µm)	Notes	Author(s)
*L. casei* 01 *L. acidophilus* LA-5 *B. lactis* BB-12	Thai herbal plants (cashew flower, yanang, pennywort, and green tea)	4 °C for 30 days	0.05% cashew flower extract increased the survival rate of *L. casei* 01 cells when compared to control and other levels (*p* < 0.05).	Not reported	The pH values of probiotic yogurts ranged between 4.45–4.48 and 4.30–4.36 at day 0 and 30, respectively. Generally, the addition of green tea extracts in encapsulating materials enhanced the stability of microbes.	[22]
*B. longum* LMG 13197	Vegetal BM 297 and inulin Freeze-drying	4° C for 6 weeks	Free cells: 2.7 logs reduction in viability during storage in yogurt. Encapsulated cells: 0.9 and 1.9 log CFU/mL reduction in viability for vegetal and vegetal-inulin encapsulated cells, respectively.	Not reported	pH of control yogurt decreased by 0.21. pH of yogurt reduced by 0.05 and 0.06 units for vegetal and vegetal-inulin encapsulated BL.	[24]
*L. paracasei* subsp. *Paracasei* E6	Whey protein isolate (WPI) and gum Arabic Complex coacervation	4 °C for 45 days	Only 0.64 and 0.22 log CFU/mL reduction in *L. paracasei* count in free cells and encapsulated, respectively.	Not reported	Post acidification rate was higher when *L. paracasei* was involved, regardless the encapsulation. >10% (w/w) coacervate inclusion, resulting weak gel structure of yogurt.	[25]
*L. acidophilus* NCFM *L. delbrueckii* subsp. *bulgaricus* *S. thermophilus*	Polymerized whey protein (PWP) Compared to Sodium alginate	4 °C for 9 weeks	PWP and alginate coated *L. acidophilus* showed higher survivability during storage. The viable count of PWP and ALG coated cells was decreased from almost 8.9 logs CFU/mL at zero time to 7.7 logs CFU/mL and 7.0 logs CFU/mL after 6 weeks, respectively.	Not reported	PWP method appeared more effective (*p* < 0.05) than SA method after the 6th week. Presence of other strains sig. decreased the viability of NCFM	[26]
*L. acidophilus* LA-5	Whey protein concentrate (WPC) and mixture of polysaccharides (sodium alginate, λ-carrageenan, inulin, lentinan, and glucose)	4 °C for 35 days	WPC: 1.5 logs cycle reduction. WPC + PS: only 0.7 logs cycle reduction over 28 days of storage. In yogurt: 1.78 and 2 logs reduction during 35 days of storage for encapsulated and free LA-5, respectively.	Not reported	Sharp decrease in pH after 2 h of fermentation (from 6.5 to 4.6) Microencapsulation sig. increase in texture and viscosity parameters of yogurt. Higher conc. Of microencapsulated cells showed the lowest syneresis (54.9%)	[28]
*L. acidophilus* ATTC-4356	Sodium alginate and carrageenan	4 °C for 18 days	Viable count of alginate and carrageenan coated cells were decreased from 9.91 logs CFU/mL and 9.89 logs CFU/mL in zero day to 8.74 logs CFU/mL and 8.39 logs CFU/mL after 28 days, respectively.	-ALG beads 714-Carrageenan 726	Encapsulated cells cause slower acidification of yogurt when compared to free cells. Sensorial attributes were sig. affected.	[30]
*Bifidobacterium* BB-12 *L. bulgaricus* (LB) *S. thermophiles* (ST)	Sweet whey (SW) and inulin (SWI) Spray drying	4 °C for 28 days	SW did not affect the viable count of *BB* during storage period compared to free cells (*p* > 0.05). (SW: initial counts were 9.20, 10.20 and 9.71 logs CFU/mL at first day, increased to be 9.32, 10.38 and 10.27 logs CFU/mL after 28 days for BB, ST and LB, respectively) Viable count of SWI coated *BB* sig. decreased (*p* < 0.05). (SWI: initial counts were 9.83, 10.23, and 9.90 logs CFU/mL at first day, then decreased to reach 9.25, 9.81, and 8.90 logs CFU/mL after 28 days for *BB*, *ST*, and *LB*, respectively).	Not reported	*LB* and *ST* count sig. affected by addition of encapsulated BB. Sig. increase in total solid in SWI and SW (*p* < 0.05) SW yogurt: sig. decrease in firmness and gumminess. SWI did not affect firmness of yogurt.	[31]
*S. thermophilus* (ST) and *L. bulgaricus* (LB) NCDC 263	Sodium alginate (ALG) Extrusion, spray drying	-	Under spray drying conditions: Survival ratio for free *LB* and *ST* was 8.27 × 10^−3^ and 2.36×10^−3^, while the encapsulated form showed higher survival of 2 logs cycle with 7.28 × 10^−1^ 2.48×10^−1^ survival ratio, respectively.	82.00–149.37	Encapsulation efficiency was the highest (~81%) when atomized pressure was 200 kPa, and 3% ALG conc. Survival ratio of *LB* under spray drying conditions was superior to that of *ST*.	[32]
*Saccharomyces cerevisiae* var. *boulardii*	Sodium alginate, extrusion	4 °C for 21 days	Encapsulated *S. boulardii* level was determined as 6.12–9.16 log CFU/g during the storage period.	Not reported	Encapsulated *S. boulardii* yogurt had a lower consistency and sensory attributes comparing to control. With no sig. difference in colour characteristics.	[33]
*L. acidophilus* LA-5	Polymerized whey protein (PWP)	4 °C for 10 weeks	Poor survivability of free *L. acidophilus* LA-5 in both cow and goat milk (~5 logs CFU/mL and 4.5 logs CFU/mL) at the zero time, respectively. Microencapsulated *LA* started with ~7.2 and 6.8 logs CFU/mL in cow and goat milk, respectively. Reached ~5.9 and 6.1 logs CFU/mL, respectively, after 10 weeks storage.	Average ~744	Encapsulation yield was 92.90 ± 3.90% LA-5 did not affect the post-acidification during storage. No sig. difference of total solids, fat and carbs. Sig. increase in protein content (*p* < 0.01) PWP: Sig. difference reported in term of firmness (311 ± 4.58 g and 287.17 ± 6.93 g) for PWP and control, respectively.	[34]
*L. lactis* Gh1	Gum Arabic Synsepalum dulcificum (miracle fruit: seed, pulp, and leaf) Spray drying	4 °C for 21 days	After 21 days of storage: -free cells: reduced from 7.36 to 5.32 logs CFU/mL in day 0 and 21 of storage, respectively. -2 logs cycle range reduction for microencapsulated cells	Not reported	The survival, encapsulation efficiency and moisture content for spray-dried *L. lactis* encapsulated with GA and MFS were 85.0%, 99.27% and 3.55%, respectively. The presence of miracle fruit parts enhanced the survivability of *L. lactis* comparing with GA alone.	[35]
*Mix: Lactobacillus delbrueckii* ssp. *bulgaricus* *(1.0%), Bifidobacterium bifidum (6.0%), Streptococcus salivarius* ssp. *thermophilus, (80.0%), Lactobacillus acidophilus*	Whey, whey protein concentrate, and sodium alginate. Spray and freeze drying	4 °C for 28 days Tested under simulated GI conditions	No significant differences (*p* > 0.05) between the counts of cells in whey-based beverages after the predicted storage period, but a significantly higher (*p* < 0.05) decrease was recorded in beverages with alginate-whey carriers prepared using spray-drying technique. Significant decrease of (*p* < 0.05) in the count of free cells compared to microencapsulated cells.	Spray drying: 5.06–7.23 Freeze drying: 2.98–3.62	The addition of protein concentrate had a positive effect on the viability of the encapsulated culture.	[37] Whey-based beverage
*L. acidophilus La-5*	Cottonseed vegetable fat Spray chilling	5 ± 1 °C for 90 days Testing under simulated GI conditions	Free cells: 0.6 logs reduction in viability during storage. Encapsulated cells: 0.5 logs reduction in viability during storage. However, for both, free and encapsulated cells, the viability still higher than 7 logs CFU/g after 90 days of storage. Significant reduction in viability of free cells when tested under GI conditions along storage duration, while encapsulated cells showed insignificant reduction. Only encapsulated cells counted higher than 6 logs CFU/g for GI testing after 60 days of storage.	78 ± 4	Adding ME probiotics did not affect pH values nor chemical compositions of cheese. Sig. increase in the MUFA and PUFA values after inoculating cheese with probiotics. Denser and more compact protein matrix of the cheese in presence of probiotics, mainly encapsulated ones, comparing to control.	[38] (requeijão cremoso processed cheese)

**Table 2:** Incorporation of microencapsulated probiotics into different types of juices and its effect on viability during storage.

Probiotic	Encapsulation Facts	Storage Conditions	Viability (At the End Storage)	CapsuleSize (µm)	Notes	Author(s)
*B.* *longum*	ALG Pineapple juice	4 °C for 45 days	Viable count of ME cells was 8.28 log CFU/g at day 45. No survival of free cells at day 30 and 45 days of storage.	Not reported.	Sensory evaluation for juice with encapsulated cells was better than that with free cells. No off-flavours nor sig. differences observed in juice with capsules.	[49]
*L.rhamnosus* GG	CHI/ALG+ inulin Apple juice	4 or 25 °C for 90 days	Free cells: Viability loss by 43% within 15 days. Survival rate for encapsulated cells was high 4.5 times more than free cells during storage.	With inulin: 1.40 ± 0.08 mmWithout inulin: 1.39 ± 0.06 mm	Presence of inulin in both temp. improve the survival of free cells, with no effect on encapsulated. Encapsulation sig. improve all sensorial attributed comparing with free cells.	[50]
*L. reuteri* NCIMB 30242	ALG and poly-L-lysine Mixed fruit	4 and 8 °C for 8 weeks.	Viability loss was lower than 1 log CFU/mL at 4 °C, with a slight increase in viability loss at 8 °C.	Not reported.	Viability losses of ME cells were not affected neither by storage time nor temperature. No observed differences in pH between free and ME	[51]
*B. animalis* BB-12	Maltodextrins and inulin Passion fruit juice	4 or 25 °C for 30 days	25 °C: Viability of PFM and PFMI reduced by 2.76 and 2.18 log CFU/mL, respectively, at day 15 of storage, while the enumeration of viability was not possible at day 30. Viability at both 4 and 25 °C after 30 days of storage was higher than therapeutic levels.	10.65 and 16.52 μm	EE of PFMI and PFI was 84.4 and 86.67%, respectively.	[52]
1. *L.* *acidophilus* 2. *L.* *plantarum* 3. *L.* *reuteri* 4. *L.* *casei* 5. *E.* *faecium*	Calcium Alginate Sour cherry juice (SCJ)	4 °C or 25 °C for 4 weeks	Viability loss was 0.7 and 2 log CFU/mL for encapsulated and free cells, respectively, at 4 °C. The viable count of encapsulated probiotics was lower than therapeutic level after 21 and 45 days at 25 and 4 °C, respectively.	Not reported	pH for encapsulated probiotic SCJ was sig. lower than those for free cells samples during storage at 4 °C. The difference in pH values amongst strains was not sig observed for first 2 weeks, after that, the pH values increased, explained by the production of amine compounds due to psychrophilic bacteria.	[53]
*Lactococcus* *lactis*	ALG, Persian Gum FOS and inulin Orange juice	4 °C for 6 weeks	Free cells: survivability decreased from 9.52 to 2.83 log CFU/mL. All microencapsulation formulations should sig. high storage stability.	ALG: 860–1130 μmALG + PG: 340–370 μm+FOS: 350–430 μm+Inulin: 460–560 μm	pH of orange juice containing encapsulated cells showed sig. lower declines from 2.93 to 2.70 during storage time, while free cells decreased pH to 2.51.	[56]
